# Effects of a Ketogenic Diet on Muscle Fatigue in Healthy, Young, Normal-Weight Women: A Randomized Controlled Feeding Trial

**DOI:** 10.3390/nu12040955

**Published:** 2020-03-30

**Authors:** Anna Sjödin, Fredrik Hellström, EwaCarin Sehlstedt, Michael Svensson, Jonas Burén

**Affiliations:** 1 Department of Food, Nutrition and Culinary Science, Umeå University, 90187 Umeå, Sweden; ewacarin.sehlstedt@umu.se (E.S.); jonas.buren@umu.se (J.B.); 2 Umeå School of Sport Sciences, Umeå University, 90187 Umeå, Sweden; 3 Department of Occupational and Public Health Sciences, Centre for Musculoskeletal Research, University of Gävle, 80176 Gävle, Sweden; fredrik.hellstrom@hig.se; 4 Department of Community Medicine and Rehabilitation, Sports Medicine Unit, Umeå University, 90187 Umeå, Sweden; michael.svensson@umu.se

**Keywords:** sports nutrition, fat adaptation, saturated fat, diet intervention, female, food, nutrition, low carbohydrate diet (LCD)

## Abstract

Ketogenic low-carbohydrate high-fat (LCHF) diets are increasingly popular in broad sections of the population. The main objective of this study was to evaluate the effects of a non-energy-restricted ketogenic LCHF diet on muscle fatigue in healthy, young, and normal-weight women. Twenty-four women were randomly allocated to a 4-week ketogenic LCHF diet followed by a 4-week control diet (a National Food Agency recommended diet), or the reverse sequence due to the crossover design. Treatment periods were separated by a 15 week washout period. Seventeen women completed the study and were included in the analyses. Treatment effects were evaluated using mixed models. The ketogenic LCHF diet had no effect on grip strength or time to fatigue, measured with handgrip test (day 24–26). However, cycling time to fatigue decreased with almost two minutes (−1.85 min 95% CI:[−2.30;−1.40]; *p* < 0.001) during incremental cycling (day 25–27), accommodated with higher ratings of perceived exertion using the Borg scale (*p* < 0.01). Participants’ own diary notes revealed experiences of muscle fatigue during daily life activities, as well as during exercise. We conclude that in young and healthy women, a ketogenic LCHF diet has an unfavorable effect on muscle fatigue and might affect perceived exertion during daily life activities.

## 1. Introduction

Often described as a multiple-cause phenomenon, fatigue can be defined as the inability to maintain power output and strength, resulting in physical and mental performance impairment [1]. As a concept, fatigue may be classified as peripheral (i.e., muscle fatigue), when changes in biochemistry occur within the skeletal muscle cell, or central, with perturbations in the central nervous system that circumscribe performance [2]. Skeletal muscle fatigue can be defined as a disruption of the force production needed to meet the demand for a given exercise intensity [3]. Undoubtedly, the underlying mechanisms in muscle fatigue development are numerous and complex [4], and development of muscle fatigue may involve many interacting factors that additionally are affected by exercise modality, inter-individual differences, and nutritional factors such as carbohydrate availability [5]. 

In recent years, ketogenic low-carbohydrate high-fat (LCHF) diets have become a popular choice of diet, not only in individuals suffering from epilepsy, diabetes, and obesity, but also in healthy individuals. The recently directed attention to the unhealthy effects of processed carbohydrates [6] has probably contributed to the popularity of ketogenic LCHF diets in broad sections of the population. Diet-induced ketosis, i.e., nutritional ketosis, usually occurs when restricting carbohydrate intake to below 20–50 g/day [7,8]. Such low carbohydrate intake causes the metabolism to adapt to produce energy from degradation of fatty acids, and possibly proteins. Volek and Phinney characterized a “well-formulated” ketogenic diet as total carbohydrate intake of less than 50 g/day, an adequate protein intake (1–1.5 g/kg/day), and fat until satiated [9]. Consequently, ketogenic diets can be described as LCHF diets with moderate protein intake.

In the area of exercise performance, there is a newfound interest in ketogenic diets. On one hand, ketogenic LCHF diets offer opportunities to induce weight loss and improve body composition, as reported recently [10,11,12]. This may increase relative, but not absolute, power due to decreased body weight, which can be important in situations when the ratio of lean to adipose body mass is crucial for performance. On the other hand, adaption to ketogenic LCHF diets also increases the capacity to utilize fat as a fuel substrate during exercise [13]. It is, of course, an appealing idea to adapt muscles to utilize the fat stores as fuel for ATP regeneration during long-term endurance exercise and, thus, slow down muscle glycogen depletion. Several studies have evaluated the effect of keto adaption on both aerobic and anaerobic performance in various athletic populations, as recently reviewed [14,15,16,17]. However, until the last five years, a vast majority of the studies have indisputable limitations in their study design, e.g., lack of randomization, too short diet intervention periods for adequate metabolic adaptations, poor control of dietary adherence, short washout periods, and few participants. In addition, most interventions have been performed in well-trained males, and it is therefore reasonable to ask whether the results of these studies apply to other, and more numerous, groups in the population [18]. For example, it is reasonable to ask whether the results of studies in male athletes apply to females, in particular to the large female population who regularly exercise, classified as “active” according to the classification system by Decroix et al. [19].

A good nutritional approach should enhance performance and delay fatigue in everyday physical activities and sessions of physical exercise. This can promote the ability to achieve and maintain physical health since epidemiological research, clinical investigations, and mechanistic studies clearly show that physical activity is essential to preventing disease, improving health, and improving quality of life [20,21]. Since a ketogenic LCHF diet alters substrate utilization patterns, it is of interest to study the effects of this diet on muscle fatigue. The aim of this study was to evaluate the effects of a non-energy-restricted ketogenic LCHF diet on muscle fatigue in a hitherto unstudied population, namely healthy, young, and normal-weight women.

## 2. Materials and Methods 

### 2.1. Study and Design Overview

**Figure 1:**
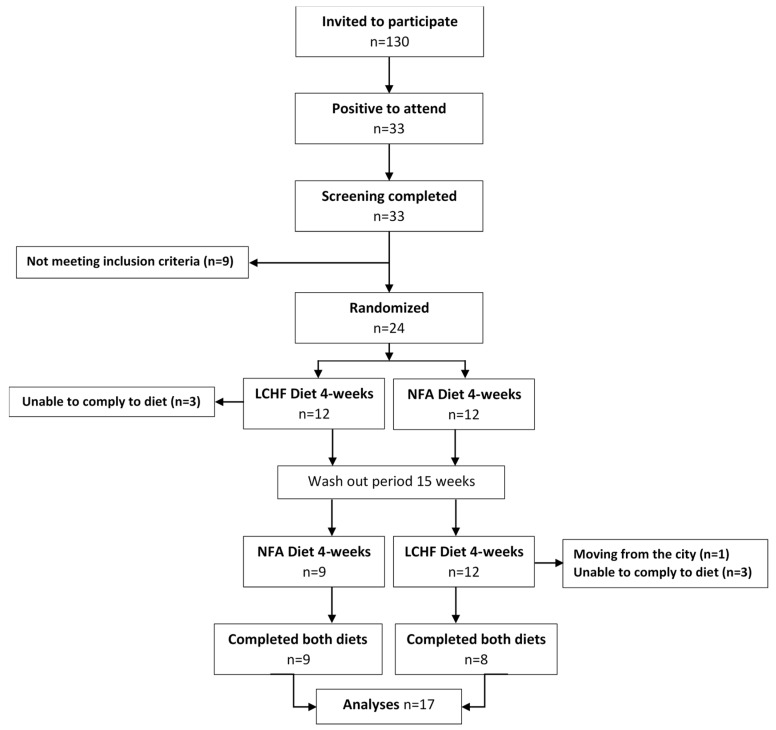
Flow of participants.

This study was a randomized controlled feeding trial. Healthy, young, normal-weight women were randomized to receive either a ketogenic LCHF diet rich in saturated fatty acids (SFA) or a control diet, a Swedish National Food Agency (NFA) recommended diet, for four weeks (Figure 1). For details, see later sections. The first four weeks of feeding trial were followed by a 15 week washout period, during which participants consumed their habitual diet. After the washout period, the groups were reversed, and each participant received the other diet for another period of four weeks, according to the crossover design. Measurements were taken prior to and at the end of each four-week diet period (in total, four examinations). Study participants were provided with all food, and the amount of energy, as well as macro- and micronutrients in the diets, was strictly defined. Participants were only allowed to eat food provided in the study and to drink water, coffee, and tea during the diet trial periods. Participants were to be weight-stable and maintain their normal level of physical activity, in order to evaluate how the diet itself affects various muscle fatigue parameters. Full details of the study protocol have been published elsewhere [22]. The clinical trial was registered at www.clinicaltrials.gov as NTC02417350.

Written informed consent was obtained from all subjects participating in the study. The study was conducted in full compliance with the Declaration of Helsinki, and the protocol of the present study was approved by the ethical review board at Umeå University, Umeå, Sweden (Dnr 2014-361-31M and Dnr 2015-45-32M). 

### 2.2. Eligibility Criteria, Participants, and Study Setting

Healthy, young (18–30 years), normal-weight (body mass index 18.5–25 kg/m^2^), and weight stable female dietetics students were recruited at Umeå University, Umeå, Sweden. Exclusion criteria were as follows: waist circumference ≥80 cm, being pregnant, diabetes, thyroid disease, prescribed medication for high cholesterol or high blood pressure, nicotine use, eating disorder, and dietary restrictions. Participants were enrolled by a research nurse and given a specific consecutive numerical code.

Thirty-three subjects were initially screened, of which 24 fulfilled the inclusion criteria, leaving 24 subjects to be randomized to one of the two study arms (Figure 1). Seventeen subjects completed the full study and were included in the analyses (for baseline characteristics, see Table 1). Two of the subjects used a bronchodilator on a daily basis (Formoterol and Budesonide), and nine used contraceptive hormones.

The study was conducted at two universities and one university hospital in Umeå, a city in northern Sweden with a population of approximately 85,000.

### 2.3. Allocation and Blinding

Eligible participants were randomly allocated in 1:1 ratio to one of the two study arms (Figure 1). Minimization was used for the allocation sequence in order to minimize imbalance of the factor weekly training sessions [23,24]. The factor “weekly training sessions” had three alternatives, with 0–2 times, 3–5 times, or 6 or more training sessions per week (the vast majority exercised 3–5 times per week). The sequence of the two diets in study arm 1 and study arm 2, respectively, was decided by a research nurse who did not participate in the analysis of data. Consequently, the researchers were blinded to group allocation until the data were analyzed and the code key was broken.

### 2.4. Study Diets

All food was provided to participants with a 14-day cyclic menu in order to have an optimal control over energy intake and diet quality. In addition, participants were only allowed to drink water, coffee, and tea. Menus and daily calories of both diets were identical for all participants. The energy need was established in a pilot study where six females, selected to resemble the experimental group, wore a SenseWear activity monitor (BodyMedia, Inc., Pittsburgh, PA, USA) on the back of the upper right arm according to the manufacturer’s recommendations. Average energy need was found to be approximately 10,000 kJ/d (2400 kcal/d), and all menus were therefore developed and nutritionally calculated based on an energy requirement of 2400 kcal/d using the dietary analysis software Dietitian Net (Kost och Näringsdata AB, Bromma, Sweden).

Both diets were identical in terms of calories, quantity of protein, and, as far as possible, recommended intake of micronutrients. The LCHF diet was a Scandinavian ketogenic LCHF diet, a diet where carbohydrates are substituted by fat, and preferably by fat from animal-based foods containing a high proportion of SFA. Typical food sources were red meat, eggs, high-fat alternatives of dairy products, and above-ground vegetables. The LCHF diet was composed of 19% of total energy intake (E%) protein, 4E% carbohydrates (daily intake should not exceed 25 g of carbohydrate excluding fiber), and 77E% fat (including 33E% saturated fat). The composition of the control diet was based on dietary guidelines presented in the Nordic Nutrition Recommendations [20], which constitutes a scientific basis for the Nordic countries to formulate their own dietary guidelines. The control diet was composed of 19E% protein, 44E% carbohydrates, and 33E% fat (including 10E% saturated fat). Participants were instructed by a research nurse to consume all food provided and to be weight stable. If they started to lose weight, they were instructed to eat extra pre-prepared snacks. There was no use of supplements and no intake of artificial sweeteners during the feeding trials.

A detailed description of food handling, food sources, nutritional composition, and photographs of the food can be found in the study protocol [22].

### 2.5. Dietary Adherence

In order to increase motivation and adherence, study participants were female dietetics students with both knowledge and interest in dietary surveys. Two female dietetic students were involved in the planning of the two diets, and great importance was given to composing menus that could be attractive to the participants in the study. At baseline assessments, the research nurse administered a daily diet and exercise logbook. Participants were instructed to note on a daily basis any deviations from the dietary protocol and to note morning weight after first void. Participants were instructed to eat extra pre-prepared snacks if losing weight. Since a ketogenic diet should lead the body to enter a state of increased fat burning and induce ketosis, participants were also instructed to have their morning urine dipped for ketones daily using a standard nitroprusside-based dipstick test (Ketostix, Bayer, Basel, Switzerland). They were instructed to read the color change after 15 seconds and to note the test result in their daily diet and exercise logbook. 

### 2.6. Outcomes and Power Calculations

As described in the study protocol [22], the primary outcome of this trial was low-density lipoprotein (LDL) cholesterol. Consequently, all outcomes in the current study i.e., handgrip time to fatigue, handgrip force, graded incremental exercise time to exhaustion, and perceived exertion are secondary outcomes. For sample size estimation, power calculations were based on the expected change in LDL cholesterol (primary outcome) as previously described [22]. An advantage in this and other studies with crossover design is that, as all participants receive the treatment under investigation, the statistical power of a crossover study is much greater than in a parallel study of equivalent size, where only half of the participants receive the treatment under investigation [25]. 

### 2.7. Daily Physical Activity Assessments 

Participants were instructed by a research nurse to maintain their normal level of physical activity. During the feeding trial periods, they were asked to daily note duration (start and stop time), intensity (scale 1–5; 1 = light activity, e.g., walking without getting sweaty; 3 = moderate physical activity, can talk at the same time; 5 = vigorous physical activity, e.g., interval training, weight lifting), and type of habitual daily physical activity and exercise training in their daily diet and exercise logbook. Based on notes in the logbook, a non-validated activity score was calculated by multiplying time x intensity for each notification, and thereafter generating a total sum (activity score) for the diet period.

### 2.8. Fasting Blood Collection and Analysis of Ketone Bodies

Fasting blood samples were obtained by a research nurse according to a standard operating protocol [22] after a 12-h overnight fast and abstinence from strenuous exercise for 24 h at day −5 to −2 (pre-diet) and at day 29 (post-diet). The first day with provided food to eat was day 1. Participants reported to the Clinical Research Center between 07:00 and 09:00 h and rested quietly for 10 min in the supine position, and blood was obtained from an antecubital vein with a 20-gauge needle and vacutainers (Becton, Dickinson and Company, Franklin Lakes, NJ, USA). Blood samples intended for serum preparation were placed at room temperature for at least 30 min and thereafter centrifuged at 1500g for 20 min at room temperature. Finally, the samples were aliquoted to 400 ul into cryogenic vials placed in iced water and immediately thereafter stored at -80 °C until use. All samples were frozen within 60 min from blood drawing.

Fasting serum β-hydroxybutyrate (the most abundant ketone body in blood) concentrations were enzymatically determined in triplicate using a colorimetric assay kit (Cayman Chemical Company, Ann Arbor, MI, USA).

### 2.9. Handgrip Force and Handgrip Time To Fatigue

Maximal voluntary isometric force (MVIF) and time to fatigue (TTF) for each participant was measured in the dominant arm before and after both diets. Assessments were conducted day −5 to −2 (pre-diet) and day 24 to 26 (post-diet). The tests were scheduled approximately two hours after lunch and performed under standardized conditions (temp 20–22 °C) on all occasions. During both the MVIF and TTF, the participants were seated in a specially designed chair with the back supported and the forearm strapped in an arm support. The position and height of the chair, table, and arm support were adjusted to achieve an elbow angle of 100° and at the same time, keeping the shoulders adducted, neutrally rotated and in a horizontal position. The forearm was in a neutral position with thumb up and the wrist in 0 degrees dorsiflexion. A foot support was used to ensure that both feet were in full contact with the floor and that the knee joint angle was approximately 90°. Each participant used the same individual settings on all four occasions. Straps were used to keep the forearm in place and to minimize compensatory movements of the upper torso during the tests.

Grip force was measured by a strain gauge dynamometer (KKM-1, Bofors, Sweden) within a handle fixed to the table. The same dynamometer was used in all trials and was calibrated before each trial. The signal from the dynamometer was sampled online at 20 Hz using a Power1401 (Cambridge Electronics Design Ltm, England) and Spike2 software (Cambridge Electronics Design Ltm, England) and stored for offline analysis.

MVIF was calculated as the average force generated during three consecutive maximal attempts with 2 sec rest in between [26,27]. The instructor orally encouraged the participant to exert maximum grip force by repeating a standardized encouraging phrase. During the TTF, the participants were given standardized instructions to do their best to maintain a steady force production using a force feedback presented on a screen in front. The screen showed a line representing 30% of initial MVIF. The test was ended when the participant was not able to maintain 20% of MVIF for a period of 5 sec. During the first minute of the test, subjects were given feedback to maintain the desired level. After the first minute, the instructor gave no feedback other than when to stop the trial. Time until task failure (i.e., beginning of the 5 sec period) was used as a measurement of handgrip endurance.

### 2.10. Graded Incremental Exercise Test, Energy Substrate Utilization, and Blood Sampling

At the Sports Medicine Unit, graded incremental exercise tests on an electronically braked bicycle (Ergomedic 839E, Monark, Vansbro, Sweden) were conducted day −5 to −2 (pre-diet) and day 25 to 27 (post-diet) for both diets. The tests were scheduled approximately one hour after lunch and performed under standardized conditions (temp 20–22 °C) on all occasions. Participants were equipped with an intravenous catheter (Optiva®2, Medex, Smiths Medical, Minnesota, USA) in a superficial forearm vein, and a transmitter heart rate monitor belt (Polar WearLinkTM31, Polar Electro Oy, Kempele, Finland) was tied around the chest. Participants started cycling at a work rate of 60 W followed by two 30 W increments every 4 min up to 120 W. At the end of each 4 min work rate period, participants were asked to rate their perceived exertion using the Borg scale [28], and blood was drawn from the vein catheter by a syringe for blood lactate measurement (Biosen C-Line Clinic, EKF-diagnostic GmbH, Barleben, Germany). After 4 min of ergometercycling at the work rate 120 W, work load was increased by 15 W/min until the participant was unable to maintain the work rate for more than 5 seconds or time of volitional exhaustion. Immediately after completed ergometer cycling, blood samples were collected from the vein catheter (post-exercise samples) for assessment of blood lactate concentrations. In addition, the participant´s perceived exertion at the time of exhaustion was noted. Throughout the ergometer cycle test respiratory gas exchange measurements of oxygen uptake (O_2_) and carbon dioxide production (CO_2_) were performed using an Oxycon Pro analysis system (Jaeger, Wuerzberg, Germany). Average values for VO_2_ and VCO_2_ (expressed as L × min−1) were calculated for each work stage. Fat and carbohydrate oxidation rates were calculated using stoichiometric equations by Péronnet and Massicotte [29]. All testing was supervised by a group of two experienced technicians.

### 2.11. Statistical Analyses

A desirable aspect of a mixed model is its ability to handle missing data. Differences in effects of diets were estimated using mixed models, with post-diet measurements (handgrip time to fatigue, handgrip force, graded incremental exercise time to exhaustion, and perceived exertion) of each diet trial as the dependent variable. Pre-diet measurements and diet were included as fixed effects, and subject was included as a random effect. To avoid cross-level bias [30], subject-averaged period baseline values were also included as fixed covariates. Analyses were performed using R version 3.5.3 (R Foundation for Statistical Computing, Vienna, Austria). The mixed models were fitted using the R function *lme* from the *nlme* package [31]. The presence of eventual carry-over effects were investigated using visual inspection of graphs and by running models where the order of diet was included as fixed effect in the models. 

Statistical analysis of weight change and activity score during the trial periods were calculated using the paired-sample *t* test using IBM´s Statistical Package for the Social Sciences package (IBM, New York, USA). 

Statistical significance was set at *p* < 0.05.

## 3. Results

### 3.1. Dietary Intake and Compliance

The participants showed good compliance to the planned diets. Dietary intake, calculated from participants’ daily notes on deviations from planned food, is presented in Table 2. Regarding macronutrient intake, protein intake did not differ between the ketogenic LCHF diet (treatment) and the NFA diet (control). As expected, the carbohydrate intake was lower and the lipid intake higher when eating the ketogenic LCHF diet compared to control diet. Regarding macro elements of importance for muscle function and fatigue, the calcium intake reached recommended daily intake for both diets, with a daily intake of 844 ± 8 mg with LCHF diet and 1029 ± 20 mg with control diet [20]. Magnesium intake did not reach the recommended daily intake of 280 mg [20] with LCHF diet (232 ± 3 mg); however, with control diet, the daily intake was 531 ± 13 mg.

Pre-diet (day −5 to day −2) fasting blood β-hydroxybutyrate concentrations were low for both ketogenic LCHF diet and control diet (0.27 ± 0.08 and 0.28 ± 0.09, respectively). Urinary ketosis was detectable after one and up to four days from the start of the LCHF diet. Once the urinary ketosis occurred, it persisted every single day throughout the LCHF feeding trial (data not shown). All women had post-diet (day 29) blood β-hydroxybutyrate concentrations >0.5 mM (1.32 ± 0.58) after eating LCHF diet for four weeks. This indicates a good compliance to the ketogenic LCHF diet throughout the diet period. As expected, there were no signs of ketosis in urine during the control diet trial (data not shown). All women had post-diet (day 29) fasting blood β-hydroxybutyrate concentrations <0.5 mM (0.28 ± 0.11) after eating a control diet for four weeks.

Weight was significantly reduced during both the LCHF and the control diet (2.8 ± 1.0 kg; *p* < 0.001, and 0.8 ± 1.4 kg; *p* < 0.05, respectively).

### 3.2. Grip Strength 

Data from the handgrip tests are presented in Table 3. Eating a ketogenic LCHF diet for 24–26 days did not induce any changes in time to fatigue, nor did the diet induce any changes in maximal force.

### 3.3. Graded Incremental Ergometer Cycling Test

**Figure 2:**
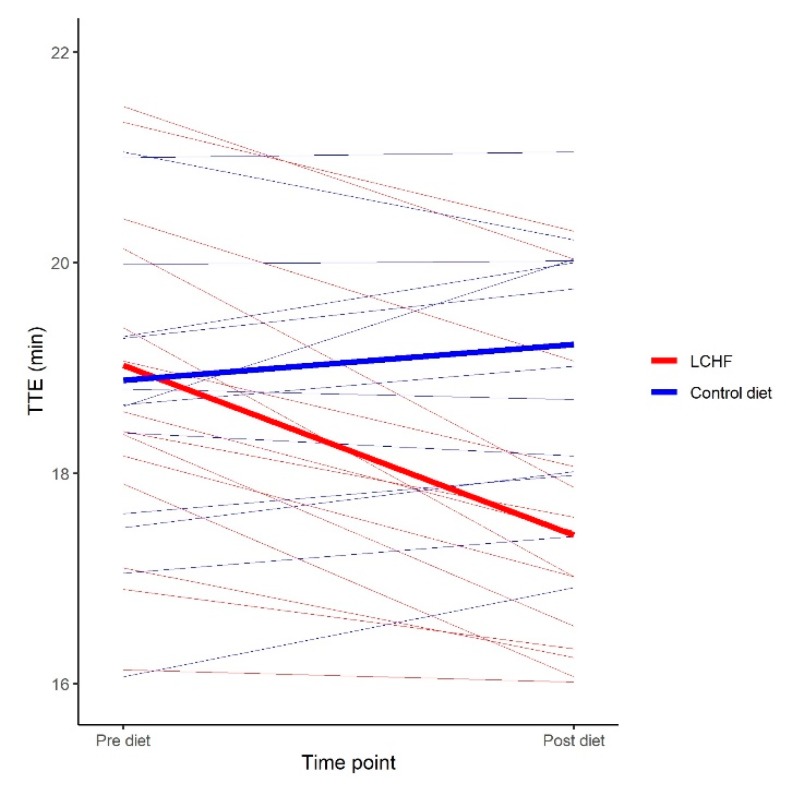
Individual changes of time to exhaustion (TTE) during a graded incremental ergometer cycling test. Pre-diet (day −5 to −2) and Post-diet (day 25–27) of the feeding trials. The ketogenic low-carbohydrate high-fat diet (LCHF) is indicated with red lines, and the Control diet is indicated with blue lines. Thick lines represent mean values for respective diet. Shown are data when both Pre-diet and Post-diet data points are available (*n* = 14 for both diets).

Data from the ergometer cycling tests are presented in Table 4. Due to upper respiratory infections, some participants could not conduct the incremental exercise test at all four occasions. Pre-LCHF and Pre-control diet each had two missing values (four participants could not conduct the tests). Post-LCHF and Post-control each had one missing value (one participant could not conduct these tests). Eating a ketogenic LCHF diet for 25–27 days induced a pronounced switch in substrate utilization. Fat oxidation increased and carbohydrate oxidation decreased throughout the submaximal range of intensities (~40%–65% VO_2max_). The ketogenic LCHF diet decreased lactate concentrations at ~40% and ~50% of VO_2max_, and at the end of the incremental exercise test. Eating a ketogenic LCHF diet mediated higher heart rate at all submaximal intensities, and decreased absolute, but not relative VO_2max_. Time to volitional exhaustion decreased (−1.85 min 95% CI:[−2.30; −1.40], *p* < 0.001, Table 4) in every participant. Individual responses in time to volitional exhaustion are shown in Figure 2. Perceived exertion was significantly higher rated at ~50% and ~ 65% of VO_2max_ after eating the ketogenic LCHF diet_,_ but did not differ to control diet at the lowest or highest work load, respectively.

### 3.4. Physical Activity During the Feeding Trials 

Notes in the logbooks revealed extensive diet-related differences in exercise-related complaints. During the ketogenic LCHF diet period, a majority of the subjects reported some kind of complaints related to exercise training. The most common among the participants (number) were impaired exercise performance (8), muscle fatigue (5), early “lactic acid” in muscles (5), and feeling tired (4). Notably, some subjects (4) noted that everyday activities, e.g., going for walks and taking the stairs, were strenuous. This was in sharp contrast to the control diet period, when there were no exercise-related complaints. On the contrary, some (*n* = 5) reported “a lot of energy” or “better than usual to exercise”. Thus, complaints related to training were more common during the ketogenic LCHF diet period. Despite diet-specific differences in exercise-related complaints, there were no differences in the activity score of the two diets (data not shown, *p* = 0.25).

## 4. Discussion

Findings from the present study are novel in that we study the effects of a ketogenic diet on muscle fatigue in a hitherto unstudied group, namely young, healthy, and normal-weight women. Earlier studies of ketogenic diet and exercise have focused mainly on athletes, and primarily male athletes [15]. 

In order to study effects of nutrition on muscle fatigue without a training-induced adaptation, our participants were instructed to keep their habitual level of physical activity throughout the study period. Based on calculations from participants’ notes in their daily diet and exercise logbooks, the amount of daily physical activity did not differ between the control and ketogenic diet period. The feeding trial also showed good compliance. All participants were keto-adapted after eating the LCHF diet for four weeks, as verified by positive testing of both urinary and blood ketones. Furthermore, respiratory exchange ratio (RER) measurements during cycling tests confirm a metabolic shift to fat oxidation. In addition, the length of the current trial is long enough for cellular adaptations to overcome the initial performance decrement associated with a ketogenic diet [32,33,34].

The ketogenic diet had no effect on peripheral muscle fatigue, measured as time to fatigue with handgrip test of low contraction intensity. Local muscle fatigue can originate from both peripheral and central factors, where handgrip test is an objective measure of peripheral fatigue. Many activities of daily living require prolonged isometric contractions with low contraction intensities [35,36]. Therefore, muscle endurance is an important aspect of physical performance. Muscle fatigue is often induced and assessed via sustained isometric maximal voluntary contraction. However, we chose to induce and assess muscle fatigue via sustained isometric voluntary contractions corresponding to 30% of individual maximal voluntary isometric force for two reasons. Firstly, fatigue assessed with maximal voluntary isometric force does not resemble muscle activities in daily life. Secondly, due to increased intramuscular pressure during sustained maximal isometric contractions, the reduced blood flow and subsequently muscle oxygenation and wash out of muscle metabolites may confound time to fatigue and conceal a possible diet-induced effect [37,38,39].

We also measured handgrip strength as maximal voluntary isometric force contraction and found no difference compared to control diet. This is a finding in contrast to an earlier study by Urbain et al. [40]. In their study of a heterogeneous group of healthy adults, a ketogenic diet resembling our diet was found to slightly increase handgrip strength. Similar to our study design, their participants’ physical activity remained unchanged during the study period, so exercise adaptations were not the explanation for the increase. Considering metabolism in skeletal muscle, it is reasonable to assume that during this short high-intensity exercise, muscle stores of adenosine triphosphate (ATP) and phosphocreatine are the dominant energy-yielding pathways [41], and therefore our finding, as we see it, is an expected outcome.

Muscle fatigue was evident in the incremental ergometer cycling test, where time to exhaustion was significantly reduced following the ketogenic LCHF diet compared to the control diet. During submaximal work (≤65% VO_2max_), a lower RER was seen when compared to the control diet, reflecting a shift in macronutrient utilization, with increased fatty acid and reduced carbohydrate oxidation after eating the ketogenic LCHF diet. Factors that affect maximal fat oxidation have been reviewed by Purdo et al. [42]. Exercise intensity is a factor that has a great impact on macronutrient utilization, where maximal rates of fat oxidation, from both extra and intramuscular fatty acids, occur at 45%–65% VO_2max_. In our study, we found that eating a ketogenic LCHF diet led to significantly increased fat oxidation at submaximal work load. Although upregulated after eating a ketogenic diet, fatty acid oxidation will never cover the high demands for the high rate of ATP re-synthesis required during high-intensity exercise [43,44]. At higher exercise intensities, the ability to exercise depends on the capacity to rapidly replace the ATP used. Re-synthesis of ATP under these conditions is met by degradation of muscle glycogen and fast anaerobic glycolysis, accompanied by the production of pyruvate and lactate, where pyruvate can be further metabolized via aerobic ATP production [45]. As the rate of glycolysis increases at increased workloads, the rate of lactate production increases [46], which is reflected by increased concentration of blood lactate [47]. In our study, we observed significantly lower peak lactate concentrations after incremental exercise. This is most likely due to impairment of the glycolytic metabolic pathway. Impairment can be due to decreased glycolytic rate as an effect of low glycogen stores in the muscle. However, despite minimal carbohydrate intake during a ketogenic diet, analysis of muscle biopsies has shown that adhering to this diet does not empty resting glycogen stores. Eating this diet for 28 days reduced glycogen stores by ~45% [48], and after 20 months, there were no differences in resting glycogen stores compared to a traditional high carbohydrate diet [49]. Impairment of the glycolytic pathway could also be due to decreased glycogenolysis and/or suppression of PDH [50], leading to earlier muscle fatigue at higher intensities. 

When discussing performance in a healthy and active population, we must keep in mind that muscle function is only one factor determining performance. Perceptions of fatigue or effort are probably important aspects for the experience of exercise, the ability to get away and exercise, and the intensity with which the workout can be carried out. The women in the current study reported increased ratings of perceived exertion at submaximal intensities of ~50%–65% VO_2max_ after eating the ketogenic LCHF diet for 25 to 27 days. Some women also spontaneously wrote down comments in their daily diet and exercise logbooks that they experienced everyday activities, such as cycling, walking, and taking the stairs, as more difficult. In line with this, in a group of healthy adults consisting of both women and men, almost half of the subjects reported their subjective physical fitness as decreased during a 6-week non-energy-restricted ketogenic diet [40]. Moreover, in a study of eighteen endurance-trained males, it was shown that the participants frequently chose a lower workload in their initial exposure to low glycogen stores [51]. On one hand, we can therefore expect a decreased exercise-induced stimulus of muscle adaptation due to exercise being performed at lower intensities after eating a ketogenic LCHF diet. Furthermore, in the literature, there is an increased recognition of the apparent linear relationship between overall physical activity and health status [52]. International physical activity guidelines recommend at least 150 minutes of moderate-intensity physical activity throughout the week [20,53]. However, current physical activity guidelines are often not met [53,54]. Fortunately, clinically relevant health benefits can be achieved by simply becoming more physically active, with greatest benefits among sedentary individuals assuming a more active lifestyle [52]. Thus, the comments from our participants regarding daily life activities are a cause for concern, and we suggest that patterns (intensities, time, etc.) of daily life activities as well as exercise should be carefully investigated in future long-time studies with ketogenic diets.

A limitation of our current study is the small sample size. Another limitation is that the menstrual cycle phase was not monitored by hormonal markers throughout the study, and nine participants used hormonal contraceptives. Steroid hormones are known to impact exercise performance, and performance tests should optimally have been conducted in a pre-specified phase [55]. Due to a relatively small number of participants, it is not feasible to conduct subgroup analyses to explore whether use of hormonal contraceptives could have an effect on our outcomes. In addition, hormonal contraceptives are a heterogeneous group of hormonal treatments. Performance tests were also conducted after a non-standardized meal (a meal within the regular menu for each diet). We chose to conduct all performance tests after lunch in a fed state, although without a standardized pre-meal due to practical circumstances. A fed state most certainly resembles conditions when most people perform physical activities, and therefore have a practical implication. In addition, testing in a fasting condition would have favored being adapted to a ketogenic diet. Another limitation is that the length of this feeding trial might be too short to allow for full keto adaptation. Finally, the fact that our participants were dietetics students with knowledge of both diet and metabolism could have affected participants’ experiences of eating both the diet under investigation and the control diet.

## 5. Conclusions

For the first time, effects of a long-term ketogenic LCHF diet on muscle fatigue in young, healthy, and normal-weight women have been investigated. The current randomized, controlled feeding trial showed that keto-adapted women switched their metabolism with pronounced increased fat utilization during submaximal work. A ketogenic diet did not affect maximal isometric force or muscle fatigue under sustained low-intensity work. However, the women experienced exercise and activities in daily life as more strenuous, and time to fatigue was shortened during incremental exercise. Being aware of these effects can help individuals make better-informed choices to maintain their activity level and stay active. It is possible that a longer keto adaptation may circumvent the negative effects of a ketogenic diet shown in this trial. Thus, further studies are warranted to investigate long-term effects of this diet on muscle fatigue.

## Figures and Tables

**Table 1:** Baseline characteristics of the subjects.

Parameters	Mean ± SD
Age (years)	23.7 ± 2.0
Body weight (kg)	61.6 ± 5.4
Body height (cm)	168 ± 5
BMI (kg/m^2^)	21.9 ± 1.3
Systolic BP (mmHg)	105 ± 8
Diastolic BP (mmHg)	64 ± 7

BMI, body mass index; BP, blood pressure.

**Table 2:** Mean daily energy and nutrient intake during 28 days of feeding trial ^1^.

Parameter	LCHF Diet		NFA Diet	
	Mean ± SD	E%	Mean ± SD	E%
Energy (kJ)	9988 ± 372		9938 ± 613	
Energy (kcal)	2387 ± 89		2375 ± 146	
Carbohydrate (g)	24 ± 4	4	259 ± 31	44
Dietary fiber (g)	9 ± 4	1	40 ± 6	3
Total fat (g)	206 ± 10	76	88 ± 17	33
-SFA (g)	88 ± 11	33	27 ± 5	10
-MUFA (g)	70 ± 8	26	33 ± 7	12
-PUFA (g)	31 ± 9	11	20 ± 6	7
Cholesterol (mg)	1070 ± 271		375 ± 104	
Protein (g)	111 ± 8	19	115 ± 11	20
Protein (g/kg body weight)	1.8 ± 0.2		1.9 ± 0.2	

^1^ Mean daily intake calculated from participants’ daily notes on deviations from planned food. LCHF Diet, ketogenic low-carbohydrate high-fat diet; NFA, a control diet based on the Nordic Nutrition Recommendations [20]; E%, percent of daily energy intake. SFA, saturated fatty acids; MUFA, mono-unsaturated fatty acids; PUFA, poly-unsaturated fatty acids.

**Table 3:** Handgrip force and handgrip time to fatigue baseline measures and treatment effects after eating a ketogenic LCHF diet for 24–26 days ^1^.

Parameter	Baseline (Mean ± SD)	Treatment Effect (95% CI)	*p*-value
HG Time to fatigue (s)	202 ± 85	−15 [−40;10]	0.26
HG MVIFmean (N)	212 ± 33	2 [−10;13]	0.77
HG MVIFpeak (N)	220 ± 35	4 [−7;16]	0.48

^1^ The treatment effect is statistically significant when *p* < 0.05. Data were analyzed using mixed models. Baseline values were assessed before start of the first diet trial (day −5 to −2) for both study arms. Abbreviations: HG, handgrip; MVIF, maximal voluntary isometric force; s, seconds; N, Newton.

**Table 4:** Graded incremental ergometer cycling test baseline measures and treatment effects after eating a ketogenic LCHF diet for 25–27 days ^1^.

Parameter	Baseline (Mean ± SD)	Treatment Effect (95% CI)	P value
TTE (min)	18.9 ± 1.6	−1.85 [−2.30; −1.40]	<0.001
VO_2max_ (L/min)	2.73 ± 0.29	−0.21 [−0.37; −0.05]	0.029
VO_2max_ (mL/kg/min)	44.24 ± 4.41	−1.49 [−3.83;0.84]	0.266
Incremental Cycling test 60 W ~40% VO_2max_			
HR (beats/min)	115 ± 11	11 [6;17]	0.004
RER	0.80 ± 0.06	−0.10 [−0.12; −0.08]	<0.001
Fat ox (g/min)	0.33 ± 0.09	0.17 [0.14;0.21]	<0.001
CHO ox (g/min)	0.37 ± 0.23	−0.45 [−0.54; −0.36]	<0.001
Lactate (mmol/L)	1.07 ± 0.31	−0.30 [−0.48; −0.12]	0.008
RPE (1-20 scale)	8.7 ± 1.2	0 [0;1]	0.631
Incremental Cycling test 90 W ~50% VO_2max_			
HR (beats/min)	135 ± 11	14 [9;18]	<0.001
RER	0.88 ± 0.05	−0.13 [−0.15; −0.12]	<0.001
Fat ox (g/min)	0.25 ± 0.12	0.32 [0.28;0.37]	<0.001
CHO ox (g/min)	1.12 ± 0.29	−0.78 [−0.87; −0.68]	<0.001
Lactate (mmol/L)	1.26 ± 0.40	−0.25 [−0.41; −0.08]	0.012
RPE (1-20 scale)	11.5 ± 1.6	2 [1;3]	0.005
Incremental Cycling test 120 W ~65% VO_2max_			
HR (beats/min)	156 ± 14	17 [10;24]	0.001
RER	0.94 ± 0.05	−0.11 [−0.13; −0.09]	<0.001
Fat ox (g/min)	0.17 ± 0.14	0.34 [0.29;0.40]	<0.001
CHO ox (g/min)	1.80 ± 0.31	−0.78 [−0.92; −0.65]	<0.001
Lactate (mmol/L)	1.81 ± 0.62	−0.13 [−0.28;0.02]	0.118
RPE (1-20 scale)	13.8 ± 1.4	2 [1;2]	<0.001
End of Incremental Cycling test			
Lactate (mmol/L)	6.72 ± 1.65	−1.71 [−2.79; −0.64]	0.012
RPE (1-20 scale)	19.5 ± 0.5	0 [0;0]	0.515

^1^ The treatment effect is statistically significant when *p* < 0.05. Data were analyzed using mixed models. Baseline values were assessed before start of the first diet trial (day −5 to −2) for both study arms. Abbreviations: TTE, time to exhaustion; VO_2max_, maximal oxygen uptake; HR, heart rate; RER, respiratory exchange ratio; Fat ox, fat oxidation; CHO ox, carbohydrate oxidation; RPE, rate of perceived exertion.
